# External validation of the VALE scoring system for hemorrhage risk in pediatric AVM patients

**DOI:** 10.1007/s10143-026-04366-y

**Published:** 2026-06-20

**Authors:** Sepide Kashefiolasl, Luciana Porto, Karsten Lachner, Michael Merker, Roland Schrewe, Susanne Schubert-Bast, Fee Keil, Vincent Prinz, Tobias Finger, Marcus Czabanka

**Affiliations:** 1 Department of Neurosurgery, University Hospital Frankfurt, Goethe University Frankfurt, Theodor-Stern-Kai 7, 60596 Frankfurt Am Main, Germany; 2 Institute of Neuroradiology, Goethe University Frankfurt, Frankfurt Am Main, Germany; 3 Department of Pediatrics, Goethe University Frankfurt, Frankfurt Am Main, Germany; 4 Epilepsy Center Frankfurt Rhine-Main, Department of Neurology, Goethe-University Frankfurt, Frankfurt Am Main, Germany

**Keywords:** Intracerebral hemorrhage, Rupture, AVM, Pediatric patients

## Abstract

Brain arteriovenous malformations (AVMs) are the leading cause of spontaneous intracranial hemorrhage in children. The recently developed VALE scoring system has demonstrated predictive value for hemorrhage risk in adult AVM patients; however, its applicability to pediatric populations remains unknown. We performed a retrospective analysis of a prospectively maintained vascular database at University Hospital Frankfurt. Pediatric patients (≤ 18 years) diagnosed with a single brain AVM between 2005 and 2023 were included. VALE scores were calculated according to the original model using ventricular system involvement, associated venous aneurysms, deep location, and exclusively deep venous drainage. Logistic regression analyses were performed to evaluate associations with hemorrhagic presentation. Discriminatory performance was assessed using receiver operating characteristic (ROC) analysis. A total of 52 pediatric AVM patients were included, of whom 31 (60%) presented with hemorrhage. Associated venous aneurysms were observed in 29% of ruptured AVMs compared with 5% of unruptured lesions and represented the only VALE component that remained significant in multivariable analysis. The complete VALE score demonstrated limited discriminatory performance, yielding an area under the ROC curve (AUC) of 0.608 (95% CI 0.454–0.762). Classification into VALE-defined risk categories showed similarly limited predictive ability (AUC 0.545, 95% CI 0.390–0.700), with no significant increase in hemorrhage risk across risk groups. This study represents the first external validation of the VALE scoring system in a pediatric AVM cohort. In our population, the VALE score demonstrated limited discriminatory performance and could not be conclusively validated. While associated venous aneurysms remained associated with hemorrhagic presentation, larger multicenter studies are required to further evaluate the applicability of the VALE score in pediatric patients and to determine whether pediatric-specific hemorrhage risk prediction models may be beneficial.

## Introduction

According to current knowledge, brain arteriovenous malformations (AVMs) are the most significant cause of spontaneous intracranial hemorrhage in children [1]. However, the dilemma between the risk of natural rupture and the adverse outcomes of intervention remains a major concern for patients with unruptured AVMs. Therefore, predicting the timing of rupture could enhance the risk–benefit analysis in the management of AVMs.

The R2eD score, developed by Feghali et al. [2], was the first scale designed to estimate the rupture risk of AVMs and was externally validated by Bird et al. [3]. This scoring system identified five risk factors based on statistical models: race (non-White), exclusive deep location, small AVM size, exclusive deep drainage, and monoarterial feeding. However, in their attempt to validate the R2eD scoring system for predicting rupture risk in pediatric AVMs, Shivani et al. [4] demonstrated poor external validity and highlighted critical differences between pediatric and adult populations, failing to capture features unique to pediatric AVMs.

Due to the cross-sectional nature of the data used to select risk factors and the lack of prospective validation of the R2eD score, Chen et al. [5] developed and validated a new scoring system. This system, known as the VALE scoring system, combines evidence-based and statistically significant imaging features to predict long-term hemorrhagic risk in patients with unruptured AVMs, using a database of 3962 adult patients. The VALE scoring system consists of four factors: ventricular system involvement (2 points), venous aneurysm (−4 points), deep location (1 point), and exclusively deep drainage (2 points). Risk levels are categorized as follows: low risk for a VALE score of less than −2, moderate risk for a score between −2 and 1, and high risk for a score greater than 1 [5].

Having been successfully validated in a prospective adult cohort [5], the VALE score has the potential to be useful in the pediatric population. However, given the unique characteristics of childhood AVMs and the higher rates of hemorrhage at presentation in pediatric patients compared to adults (70–80% [6–8] vs. 45–60% [3, 9, 10]), the new scoring system predicting hemorrhagic risk in adult AVMs needs independent validation in children before it can be widely adopted for use in pediatric patients.

Consequently, we aimed to provide the first report of external validation of the VALE scoring system specifically for the pediatric population, evaluating its ability to predict hemorrhagic risk in pediatric patients, which is crucial for neurosurgical and clinical care.

## Methods

### Study population

A prospectively collected vascular database at University Hospital Frankfurt was retrospectively reviewed for pediatric patients (aged ≤ 18 years) diagnosed with arteriovenous malformations (AVMs) between 2005 and 2023. This database was established to compile all vascular cases evaluated or treated by the neurosurgical team at University Hospital Frankfurt.

As a tertiary referral center, our institution primarily receives patients referred for specialized neurovascular evaluation and treatment.

In total, 88 pediatric patients with AVMs were identified. Patients with multiple AVMs on imaging studies, those diagnosed with spinal AVMs, and individuals with incomplete electronic medical records were excluded. In the final study cohort, each patient’s imaging and clinical data were reviewed and assigned scores as follows: 0 or 2 points for ventricular system involvement, 0 or − 4 points for the presence of a venous aneurysm (defined according to the original VALE study and distinct from arterial or intranidal aneurysms), 0 or 1 point for deep location, and 0 or 2 points for exclusively deep venous drainage. The total score and hemorrhage risk were calculated according to the original report by Chen et al. Low risk was defined as a VALE score < − 2, moderate risk as a VALE score between − 2 and 1, and high risk as a VALE score > 1.5.

Imaging data were primarily obtained from catheter-based digital subtraction angiography, when available, to allow detailed assessment of vascular angioarchitecture. When angiographic data were unavailable, computed tomography angiography (CTA) or magnetic resonance imaging/magnetic resonance angiography (MRI/MRA) were used. Patients were scored for each category based on the appropriate radiographic criteria. All imaging studies were reviewed by experienced neuroradiologists involved in the care of pediatric AVM patients.

When available, digital subtraction angiography was preferentially used to assess AVM angioarchitecture and determine the individual VALE score components.

### Ethics approval and consent to participate

This retrospective clinical study was approved by the Ethics Commission of the Medical Faculty of Goethe University Frankfurt am Main, Germany (Approval Number: 2025–2270) and was conducted in accordance with the ethical standards of the institutional and regional research committee as well as with the Declaration of Helsinki.

Due to the retrospective nature of the study and the use of anonymized data, informed consent from patients or legal guardians was waived by the ethics committee.

Human Ethics and Consent to Participate declarations: not applicable.

### Funding

This research received no external funding.

### Clinical trial registration

Clinical trial number: not applicable.

This study is a retrospective observational analysis and does not meet the criteria of a clinical trial.

### Statistical analysis

Statistical analyses were performed using IBM SPSS Statistics version 29 (IBM Corp., Armonk, NY, USA). Descriptive statistics were used to summarize demographic and clinical characteristics. Categorical variables were analyzed using Fisher’s exact test or the chi-square test, as appropriate. A *p*-value < 0.05 was considered statistically significant.

Univariate binary logistic regression analyses were conducted to calculate odds ratios (ORs) with corresponding 95% confidence intervals (CIs) for each component of the VALE score. Multivariate logistic regression analysis was subsequently performed to identify independent predictors of AVM rupture. Variables included in the multivariate model were derived from the original VALE scoring system (V, A, L, and E). A stepwise backward elimination approach was applied, with a significance threshold of *p* < 0.05.

The final multivariable model and the complete VALE score were used to calculate the area under the curve (AUC) of the receiver operating characteristic (ROC) curve to assess predictive performance.

## Results

### Study population

**Fig. 1:**
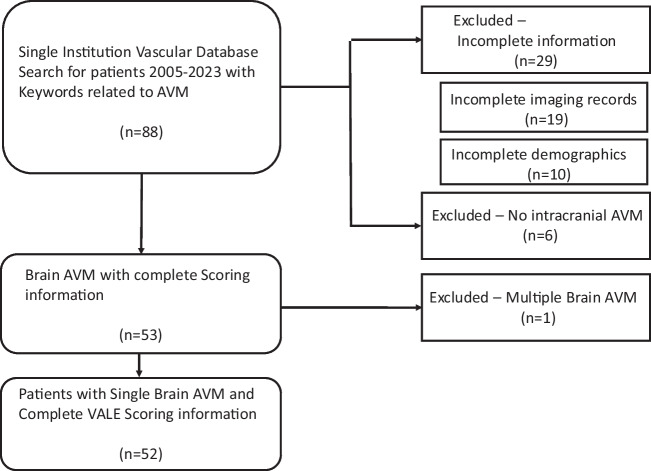
Study flowchart illustrating patient selection for external validation of the VALE scoring system in pediatric brain arteriovenous malformations (AVMs). A total of 88 pediatric AVM patients were identified from the institutional vascular database between 2005 and 2023. After exclusion of patients with multiple AVMs, spinal AVMs, and incomplete clinical records, 52 patients with a single brain AVM were included in the final analysis

We evaluated a total of 52 pediatric patients with single brain arteriovenous malformations (AVMs) who met the inclusion criteria, as illustrated in Fig. 1. The cohort displayed no significant sex predominance, with females accounting for 52% of the population. The mean age at diagnosis was 9.46 ± 4.8 years, with no significant difference in age observed between patients with ruptured and unruptured AVMs (*p* = 0.67). (Table 1).

**Table 1:** Demographics and characteristics of ruptured and unruptured pediatric AVMs

	Total *n* = 52	Rupture Yes, *n* = 31	No, *n* = 21	*P* Value
Sex				
Female	27 (52%)	17 (55%)	10 (48%)	0,78
Male	25 (48%)	14 (45%)	11 (52%)	
Mean age at diagnosis, Yrs	9,46 ± 4,8	9,19 ± 5	9,86 ± 4,48	0,67
Intervention	37 (71%)	31 (100%)	6 (29%)	
**Surgery**	**12 (32%)**	**11 (35%)**	**1 (17%)**	**0,017**
Endovascular treatment	12 (32%)	10 (32%)	2 (33%)	0,093
Radiosurgery	8 (22%)	6 (19%)	2 (33%)	0,445
Combined treatment	5 (14%)	4 (13%)	1 (17%)	0,637
AVM nidus size, cm				
Small < 3	20 (38%)	11 (35%)	9 (43%)	0,772
Large ≥ 3	32 (62%)	20 (65%)	12 (57%)	
Location				
Frontal	9 (17%)	3 (10%)	6 (29%)	0,133
Occipital	9 (17%)	7 (23%)	2 (10%)	0,283
parietal	12 (23%)	8 (26%)	4 (19%)	0,741
temporal	3 (6%)	3 (10%)	0 (0%)	0,264
Deep location: thalamus	6 (12%)	3 (10%)	3 (14%)	0,675
Basal ganglia	4 (8%)	2 (6%)	2 (10%)	> 0,999
Brainstem	2 (4%)	1 (3%)	1 (5%)	> 0,999
cerebellum	5 (10%)	3 (10%)	2 (10%)	> 0,999
intraventricular	2 (4%)	1 (3%)	1 (5%)	> 0,999
Ventricular system involvement	13 (25%)	7 (23%)	6 (29%)	0,747
**Associated venous aneurysm**	**10 (19%)**	**9 (29%)**	**1 (5%)**	**0,036**
Deep venous drainage	37 (71%)	23 (74%)	14 (67%)	0,756
Spetzler Martin grade				
I	3 (6%)	1 (3%)	2 (10%)	0,558
II	22 (42%)	13 (42%)	9 (43%)	> 0,999
III	24 (46%)	15 (48%)	9 (43%)	0,781
IV	3 (6%)	2 (6%)	1 (5%)	> 0,999
V	0 (0%)	0 (0%)	0 (0%)	> 0,999
Leading presenting symptoms				
Headache	19 (37%)	12 (39%)	7 (33%)	0,774
Cranial nerve disorders	9 (17%)	5 (16%)	4 (19%)	> 0,999
Sensomotoric deficits	11 (21%)	7 (23%)	4 (19%)	> 0,999
Seizure	8 (15%)	5 (16%)	3 (14%)	> 0,999
Hydrocephalus	3 (6%)	1 (3%)	2 (10%)	0,558
Asymptomatic	2 (4%)	1 (3%)	1 (5%)	> 0,999

Values are presented as number (%) of patients or mean ± SD unless otherwise indicated. Boldface type indicates statistical significance. *p < 0,05. Mean with 95% CI

In this cohort, 60% (*n* = 31) of the patients presented with AVM rupture. Surgical (*n* = 12, 32%) and endovascular (*n* = 12, 32%) interventions were the most common treatment modalities. Notably, surgical intervention was significantly more frequent in ruptured cases (11/12, 92%, *p* < 0.05). Among the patients who received endovascular treatment, 10 out of 12 had ruptured AVMs. Additionally, only 6 patients (12%) underwent intervention prior to rupture. (Table 1).

The predominant location of AVMs in the cohort was in deep structures, including the thalamus, basal ganglia, brainstem, cerebellum, and intraventricular regions (*n* = 19, 37%). This was followed by the parietal (*n* = 12, 23%), frontal (*n* = 9, 17%) and occipital (*n* = 9, 17%) regions. (Table 1).

Among patients with ruptured AVMs, the majority had an AVM size ≥ 3 cm (*n* = 20, 65%) and demonstrated deep venous drainage (*n* = 23, 74%). Ventricular system involvement was noted in 23% (*n* = 7) of the ruptured group on MRI, with no significant differences compared to the unruptured group. The most common locations for ruptured AVMs were deep structures (*n* = 10, 32%), followed by the parietal (*n* = 8, 26%) and occipital (*n* = 7, 23%) regions. In the presence of associated venous aneurysms, 9 out of 10 patients were in the ruptured group. The leading presenting symptom in the ruptured cohort was headache (*n* = 12, 39%), followed by sensorimotor deficits (*n* = 7, 23%). Other symptoms included cranial nerve disorders (*n* = 5, 16%), seizure (*n* = 5, 16%), and hydrocephalus (*n* = 1, 3%). One patient was asymptomatic (3%), defined as the absence of all other symptoms. (Table 1).

In the unruptured AVM group, the majority also had an AVM size ≥ 3 cm (*n* = 12, 57%) and exhibited exclusive deep drainage (*n* = 14, 67%). Only 1 out of 21 patients (5%) had an associated venous aneurysm, and 6 out of 21 (29%) showed ventricular system involvement. The most common location for unruptured AVMs was exclusively deep location (*n* = 9, 43%), followed by frontal (*n* = 6, 29%) and parietal (*n* = 4, 19%) regions. In this cohort (*n* = 21), the most prevalent symptom was headache (*n* = 7, 33%), followed by cranial nerve disorders (*n* = 4, 19%) and sensorimotor deficits (*n* = 4, 19%). Differences in symptom presentation between the ruptured and unruptured groups were not statistically significant. (Table 1).

Regarding the VALE score system, the presence of an associated venous aneurysm was identified as a significant risk factor for AVM rupture in our cohort (Table 1). Univariate analysis of demographic data and each VALE score component revealed no significant associations with rupture onset for the four included factors (ventricular system involvement, venous aneurysm, deep location, and exclusively deep venous drainage). However, in multivariate backward logistic regression analysis, the presence of an associated venous aneurysm emerged as statistically significant in the final model (Table 2). The AUC for the model based solely on the associated venous aneurysm score was 0.621 (95% CI 0.47–0.773).

**Table 2:** Univariate and multivariate analysis of VALE score components on rupture presentation

Component	Ruptured	Univariable OR (95% CI; p value)	Multivariable OR (95% CI; p value)
**V**: **V**entricular system involvement			
Yes	7/13 (54%)	0,73 (0,20–2,59; 0,63)	1,70 (0,32–9; 0,54)
No	6/39 (15%)		
**A**: Venous **A**neurysm			
Yes	9/10 (90%)	8,14 (0,94–70,41; 0,057)	**17,19 (1,53–193,41; 0,021)**
No	1/42 (3%)		
**L**: Deep **L**ocation			
Yes	11/22 (50%)	0,61 (0,20–1,87; 0,38)	0,31 (0,07–1,46; 0,14)
No	11/30 (37%)		
**E**: **E**xclusively deep drainage			
Yes	23/37 (62%)	1,48 (0,44–5,03; 0,53)	4,54 (0,82–25,14; 0,08)
No	14/15 (93%)		

Values are presented as number of ruptured AVMs/total number of AVMs (%) unless otherwise indicated. *p < 0,05. Mean with 95% CI

### Score performance

**Fig. 2:**
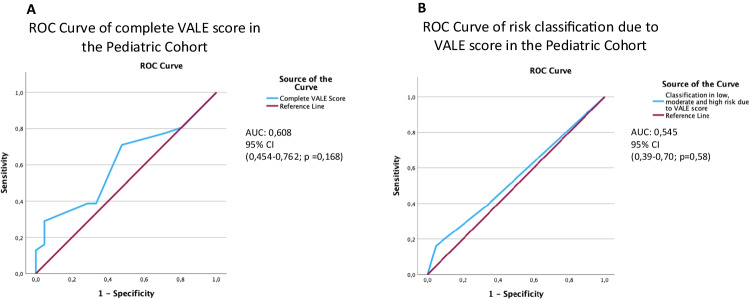
Receiver operating characteristic (ROC) curve analysis of the VALE scoring system in the pediatric AVM cohort. (A) ROC curve demonstrating the discriminatory performance of the complete VALE score for predicting hemorrhagic presentation (AUC 0.608, 95% CI 0.454–0.762). (B) ROC curve demonstrating the discriminatory performance of VALE risk-group classification (low, moderate, and high risk) for predicting hemorrhagic presentation (AUC 0.545, 95% CI 0.390–0.700)

In our cohort of 52 patients with complete VALE score data, the distribution of scores was as follows: −4 in 4 patients (8%), −3 in 2 patients (4%), −2 in 2 patients (4%), −1 in 2 patients (4%), 0 in 8 patients (15%), 1 in 1 patient (2%), 2 in 13 patients (25%), 3 in 7 patients (13%), 4 in 3 patients (6%), and 5 in 10 patients (19%). The complete model for the VALE score demonstrated an area under the curve (AUC) of 0.608 (95% CI 0.454–0.762) (Fig. 2A).

The proportion of patients presenting with rupture exhibited a left-skewed distribution. Notably, patients with a VALE score of 2 had the highest proportion of ruptured presentations (32%), followed by those with a score of 5 (19%). Both groups were categorized as high rupture risk (defined as a VALE score greater than 1). Conversely, patients with a VALE score of −4, representing 13% of the cohort, were classified as low risk.

We evaluated the classification of risk (low, moderate, and high) based on the VALE score using a model discrimination approach with the ROC curve. This analysis yielded an AUC of 0.545 (95% CI 0.39–0.70) (Fig. 2B). Consistent with the findings from the VALE score, there was no significant increase in hemorrhage risk among patients categorized in the "high risk" group (Table 3).

**Table 3:** Hemorrhage risk according to VALE score in the pediatric cohort

Risk group	Rupture Yes, *n* = 31	No, *n* = 21	OR, 95% CI	P Value*
Low	5 (16%)	1 (5%)	3,85, 0,43–47,29	0,382
Moderate	7 (23%)	6 (29%)	0,729, 0,19–2,47	0,747
High	19 (61%)	14 (66%)	0,792, 0,25–2,62	0,774

Values are presented as number (%) of patients unless otherwise indicated. *p < 0,05. Mean with 95% CI

## Discussion

The rupture of brain arteriovenous malformations (AVMs) represents a significant complication, leading to intracranial hemorrhage that notably contributes to morbidity and mortality [11]. In the management of patients with unruptured brain AVMs, especially within pediatric populations, there is an ongoing challenge in finding the optimal risk–benefit balance between invasive interventions and vigilant monitoring [12]. AVMs are the primary cause of spontaneous intraparenchymal hemorrhage in children, excluding cases linked to prematurity [13–15]. Given the unique presentation patterns and differing hemorrhage risk factors between adults and children, it is crucial to specifically assess the rupture risk in the pediatric demographic.

Rangwala et al. [4] conducted the first external validation of the R2eD AVM scoring system in a pediatric cohort. Their analysis revealed significant discrepancies between pediatric and adult populations, including a markedly higher incidence of hemorrhagic presentation in children and the absence of racial associations with AVM rupture risk. These findings highlight the limitations of adult-derived models and emphasize the need for risk stratification tools specifically adapted to the pediatric population.

Beyond the current lack of pediatric-specific scoring systems, it is crucial to consider the cumulative lifetime hemorrhage risk when managing AVMs in children. As proposed by Glazener et al. [16], a simplified estimation—Hemorrhage risk ≈ 105 minus the patient’s age in years—underscores how even a seemingly low annual rupture risk can become clinically significant across a child’s lifetime. This reinforces the importance of developing risk models that extend beyond short-term risk prediction and better reflect the lifelong burden of disease.

In this context, we advocate for the development of a modified rupture risk score tailored to pediatric AVM patients. Although the Spetzler–Martin grading system remains an important tool for operative planning, it does not quantify rupture risk or integrate pediatric-specific anatomical or temporal factors. A revised model should incorporate morphological characteristics such as exclusive deep venous drainage, presence of associated venous aneurysms, and age-adjusted cumulative risk exposure. Such a tool would not only improve individualized risk prediction but also inform therapeutic decision-making. Multicenter collaborations will be critical to successfully design and validate this framework.

### Performance of the VALE score in a pediatric cohort

Our study aimed to evaluate the applicability of the VALE scoring system in pediatric patients. In our cohort, the VALE score demonstrated limited discriminatory performance, with an AUC of 0.608. While this finding may suggest differences in the behavior of individual VALE components between pediatric and adult populations, the limited sample size of the present study precludes definitive conclusions regarding the performance of the score in children.

Notably, 39% of patients classified as low- to moderate-risk according to the VALE score presented with hemorrhage. Although this finding may indicate reduced clinical utility of the score in our cohort, larger multicenter studies are required to determine whether this observation reflects true differences in pediatric AVM biology or the limited statistical power of the present study.

### Component V of the VALE score (Ventricular System Involvement) and Its Diminished Impact on Pediatric AVM Rupture Risk

Regarding Component V of the VALE score—ventricular system involvement—we observed a reduced impact on rupture risk in pediatric patients. The VALE score assigns considerable weight to this factor, reflecting earlier studies that indicated an elevated risk of hemorrhage associated with AVMs located near the ventricular system [5, 17]. However, in our cohort of 13 patients (25%) with ventricular system involvement, only 54% experienced a rupture, compared to 46% of those with unruptured AVMs. Our univariate and multivariate analyses suggest that the influence of ventricular system involvement on rupture risk may be less significant in pediatric patients. This finding warrants further investigation to better understand the relationship between anatomical proximity to the ventricular system and the risk of bleeding in children.

### Component A of the VALE score: associated venous aneurysms and hemorrhagic presentation in pediatric AVMs

The A component of the VALE score specifically refers to associated venous aneurysms rather than arterial feeder aneurysms or intranidal aneurysms. Consequently, our interpretation focused on this distinct angioarchitectural feature, and comparisons with studies evaluating aneurysms as a heterogeneous entity should be made with caution.

In our cohort, associated venous aneurysms demonstrated the strongest association with hemorrhagic presentation and represented the only VALE component that remained statistically significant in multivariable analysis in this cohort. Venous aneurysms were identified in 29% of ruptured AVMs compared with only 5% of unruptured lesions. Although the absolute number of affected patients was small, this observation is consistent with the importance attributed to venous aneurysms in the original VALE model.

The underlying mechanisms linking venous aneurysms to hemorrhagic presentation remain incompletely understood. However, venous aneurysms may reflect altered venous hemodynamics and localized venous wall stress within the AVM drainage system. In our series, their presence was predominantly observed in patients presenting with hemorrhage, suggesting that this angioarchitectural feature may remain clinically relevant when assessing rupture risk in pediatric AVMs. Nevertheless, given the limited cohort size, these findings should be interpreted cautiously and require confirmation in larger multicenter studies.

### Component L of the VALE score: AVM locations and their associated rupture risk in pediatric patients

In their foundational study, Chen et al. [5] characterized the VALE component L (exclusive deep location) as encompassing arteriovenous malformations (AVMs) located in the thalamus, basal ganglia, brainstem, and cerebellum. In our investigation, we observed that 32% of patients with deep AVM locations experienced hemorrhage; however, this finding did not reach statistical significance. Across multiple studies [2, 4, 5], the thalamus (*n* = 3; 10%) and cerebellum (*n* = 3; 10%) were notably overrepresented in the ruptured AVM group compared to their overall spatial distribution.

In our pediatric cohort with ruptured AVMs, the most prevalent locations were deep AVMs (32%), followed by parietal (26%) and occipital (23%). In contrast, the most common ruptured AVM locations in both original and external validation adult groups were also deep (31.7%), temporal (27%), and parietal (23.8%) AVMs, respectively [5].

Additionally, another study indicated that temporal lobe AVMs have a higher likelihood of presenting with hemorrhage compared to those in the parietal lobe [18]. The literature presents mixed findings regarding the correlation between AVM location and hemorrhage risk in children. Some studies suggest that infratentorial AVMs are more prone to rupture [19], while others indicate a greater risk associated with supratentorial locations [20]. This aspect of the VALE score necessitates further investigation, particularly to explore potential variations in weight patterns for the pediatric population when assessing the risk of AVM hemorrhage.

### Component E of the VALE score: different associations of venous drainage and rupture risk in children and adults

Exclusive deep venous drainage has been extensively investigated as a risk factor for AVM hemorrhage in children [20–25] and is more frequently observed in younger patients [11]. In our study, the prevalence of exclusively deep venous drainage in the rupture group was significantly higher at 74%, compared to 32.9% in the external validation study [5]. However, a substantial number of pediatric patients in our cohort who did not experience rupture also exhibited deep venous drainage (67%). Consequently, the association between deep venous drainage and hemorrhage risk was not statistically significant in both univariate and multivariate analyses. This indicates that the E component may not be a reliable predictor for assessing hemorrhage risk in pediatric AVMs.

### Study limitations

The patient population in this study consists of a medium-sized cohort of pediatric AVM patients, totaling 52 individuals. While this cohort size is smaller compared to the original cohort of 3962 patients from Chen et al. [5] or the 377 patients in the multicenter external validation cohort, it may still provide relevant insights into the validity of the VALE score [26, 27]. However, the limited cohort size raises questions about the statistical rigor necessary for robust validation. Notably, only one component of the VALE score— the presence of an associated venous aneurysm—demonstrated significant relevance for evaluating rupture risk in pediatric patients, which further questions the validation study’s statistical strength.

Additional limitations inherent in a retrospective review include the potential for missing records or imaging data.

Furthermore, because patient identification was based on a prospectively maintained institutional vascular database, cases of fatal hemorrhage occurring prior to referral or transfer to our institution may not have been captured, which could have resulted in an underrepresentation of the most severe presentations.

Moreover, due to the retrospective nature of the study, the cohort predominantly includes patients who presented with hemorrhage and underwent acute treatment, which limits the inclusion of incidentally discovered AVMs. To address these limitations and enhance understanding of pediatric AVM rupture risk, multi-institutional collaboration across various geographic regions is essential. The smaller sample size also contributed to the absence of significant differences in rupture rates among AVMs categorized into low, moderate, and high-risk groups according to the established VALE score, a finding that contrasts with results from adult studies.^5^ While our highest rupture rate was observed in the high-risk group (see Table 3), this may reflect statistical variability due to the smaller sample sizes at both lower and higher risk scores, rather than indicating a true difference in rupture risk.

Furthermore, not all patients had complete DSA datasets available. Although DSA was preferentially used whenever available, CTA and MRI/MRA were required in selected cases. This may have introduced imaging-related heterogeneity in the assessment of VALE score components (Table 4).

**Table 4:** Hemorrhage risk according to Spetzler-Martin score in the pediatric cohort

Risk group	Rupture Yes, *n* = 31	No, *n* = 21	OR, 95% CI	*P* Value*
I	1 (3%)	2 (10%)	0.28 (0.02–3.65)	0.558
II	13 (42%)	9 (43%)	0.97 (0.31–3.02)	> 0.999
III	15 (48%)	9 (43%)	1.20 (0.39–3.72)	0.781
IV	2 (6%)	1 (5%)	1.36 (0.11–17.04)	> 0.999
V	0 (0%)	0 (0%)	-	> 0.999

Values are presented as number (%) of patients unless otherwise indicated. *p < 0,05. Mean with 95% CI

### Future directions

Future research should focus on identifying demographic and radiographic characteristics associated with hemorrhagic presentation in pediatric AVMs. In the present cohort, associated venous aneurysms were the only VALE component that remained significantly associated with rupture in multivariable analysis. Given the limited sample size, this observation should be interpreted cautiously and requires confirmation in larger multicenter cohorts.

Future collaborative studies may help determine whether pediatric-specific modifications of existing hemorrhage prediction models are warranted and whether such models could improve individualized risk assessment in children with AVMs.

## Conclusions

This study represents the first external evaluation of the VALE scoring system in a pediatric AVM cohort. The VALE score demonstrated limited discriminatory performance in our cohort and therefore could not be conclusively validated in this pediatric population. However, given the relatively small sample size and single-center design, these findings should be interpreted with caution.

Our results suggest that the performance of the VALE score in pediatric patients may differ from that reported in adult populations. Larger multicenter studies are required to further assess the applicability of the VALE score and to determine whether pediatric-specific risk prediction models may be beneficial.

## Data Availability

No datasets were generated or analysed during the current study.
